# New Evidence on a Distinction between Aβ40 and Aβ42 Amyloids: Thioflavin T Binding Modes, Clustering Tendency, Degradation Resistance, and Cross-Seeding

**DOI:** 10.3390/ijms23105513

**Published:** 2022-05-15

**Authors:** Anna I. Sulatskaya, Georgy N. Rychkov, Maksim I. Sulatsky, Ekaterina V. Mikhailova, Nadezhda M. Melnikova, Veronika S. Andozhskaya, Irina M. Kuznetsova, Konstantin K. Turoverov

**Affiliations:** 1 Laboratory of Structural Dynamics, Stability, and Folding of Proteins, Institute of Cytology, Russian Academy of Sciences, 4 Tikhoretsky Ave., 194064 St. Petersburg, Russia; ansul@mail.ru (A.I.S.); 4evamkh@gmail.com (E.V.M.); melnikova07nm@gmail.com (N.M.M.); veronika.andozhskaya@gmail.com (V.S.A.); imk@incras.ru (I.M.K.); 2 Laboratory of Molecular and Cellular Biophysics, Petersburg Nuclear Physics Institute Named by B.P. Konstantinov of National Research Center «Kurchatov Institute», Mkr. Orlova Roshcha 1, 188300 Gatchina, Russia; georgy-rychkov@yandex.ru; 3 Institute of Biomedical Systems and Biotechnology, Peter the Great St. Petersburg Polytechnic University, Polytechnicheskaya 29, 195251 St. Petersburg, Russia; 4 Laboratory of Cell Morphology, Institute of Cytology, Russian Academy of Sciences, 4 Tikhoretsky Ave., 194064 St. Petersburg, Russia; m_sulatsky@mail.ru

**Keywords:** Alzheimer’s disease, amyloid fibrils, Abeta peptide, seeding and cross-seeding, structure, stability and cytotoxicity, fluorescent probe thioflavin T, thioflavin T binding sites

## Abstract

The relative abundance of two main Abeta-peptide types with different lengths, Aβ40 and Aβ42, determines the severity of the Alzheimer’s disease progression. However, the factors responsible for different behavior patterns of these peptides in the amyloidogenesis process remain unknown. In this comprehensive study, new evidence on Aβ40 and Aβ42 amyloid polymorphism was obtained using a wide range of experimental approaches, including custom-designed approaches. We have for the first time determined the number of modes of thioflavin T (ThT) binding to Aβ40 and Aβ42 fibrils and their binding parameters using a specially developed approach based on the use of equilibrium microdialysis, which makes it possible to distinguish between the concentration of the injected dye and the concentration of dye bound to fibrils. The binding sites of one of these modes located at the junction of adjacent fibrillar filaments were predicted by molecular modeling techniques. We assumed that the sites of the additional mode of ThT-Aβ42 amyloid binding observed experimentally (which are not found in the case of Aβ40 fibrils) are localized in amyloid clots, and the number of these sites could be used for estimation of the level of fiber clustering. We have shown the high tendency of Aβ42 fibers to form large clots compared to Aβ40 fibrils. It is probable that this largely determines the high resistance of Aβ42 amyloids to destabilizing effects (denaturants, ionic detergents, ultrasonication) and their explicit cytotoxic effect, which we have shown. Remarkably, cross-seeding of Aβ40 fibrillogenesis using the preformed Aβ42 fibrils changes the morphology and increases the stability and cytotoxicity of Aβ40 fibrils. The differences in the tendency to cluster and resistance to external factors of Aβ40 and Aβ42 fibrils revealed here may be related to the distinct role they play in the deposition of amyloids and, therefore, differences in pathogenicity in Alzheimer’s disease.

## 1. Introduction

The formation and accumulation of protein aggregates, amyloid fibrils, and their precursors (toxic amyloid oligomers) in the tissues and organs of patients is associated with many serious diseases, such as neurodegenerative Alzheimer’s and Parkinson’s diseases, cataracts, malignant myeloma, prion diseases, and type II diabetes (see, e.g., [1,2,3,4,5,6]). Such diseases were previously considered rare, but with an increase in life expectancy due to an improvement in quality and healthcare advances, they began to acquire the scope of epidemics among the elderly [7,8,9,10,11,12]. In particular, Alzheimer’s disease is called the “epidemic of the 21st century” and is very likely to become the most prevalent disease in the world [13].

A hallmark of Alzheimer’s disease is the accumulation in the patient brain of amyloid plaques formed from the Abeta peptide, which consists of 42 amino acid residues (Aβ42) [14,15]. However, there are other varieties of this peptide, which are normally formed from a transmembrane protein, the precursor of beta-amyloid (APP) [16]. In particular, in the cerebrospinal fluid of healthy people (not suffering from Alzheimer’s disease), the ratio of the main Abeta peptides is estimated approximately as 50% Aβ40, 16% Aβ38, and 10% Aβ42 (Scheme S1). According to literature, these peptides have neuroprotective and neurotrophic actions in trophic deprived conditions [16,17,18,19]. At the same time, the reasons for the differences in their pathogenicity in Alzheimer’s disease remain not fully understood.

According to modern concepts, differences in the cytotoxicity of amyloid fibrils can be determined by their structure, size, and stability [20,21,22,23]. Results of in vitro studies confirm that the amyloid fibrils formed from various variants of Abeta peptides are significantly different. In particular, in [24], the key role of N-terminal amino acid residues in the stability of Abeta-peptide amyloid fibrils was shown by atomistic molecular dynamics simulations. In addition, the results of NMR spectroscopy showed the differences in the structural organization of fibril fragments formed from Aβ40 and Aβ42 [25,26] (PDB codes: 2M4J and 2NAO, respectively).

Despite numerous studies aimed at comparing the characteristics of Aβ40 and Aβ42 amyloid fibrils, the identification of the relationships between the structure, properties, and cytotoxicity of these amyloids remains relevant. It should be noted that different experimental studies of the fibrils formed from Abeta-peptides often have a narrow task of evaluating their individual characteristics. As a result, the various conditions of Abeta-peptide fibrillogenesis (as well as other amyloidogenic proteins) leading to polymorphism of the forming aggregates make it difficult to analyze the aforementioned associations systematically. In this work, we (1) carried out a comprehensive comparative study of the morphology, secondary structure, and stability of amyloid fibrils formed from recombinant Aβ40 and Aβ42 peptides using a wide range of physicochemical approaches and molecular modeling techniques; (2) used specially developed methods [27,28] to analyze various modes of their interaction with fluorescent probe thioflavin T (ThT), which is the “gold standard” for diagnosing of amyloid fibrils [29,30,31,32]; and (3) investigated the amyloid ability of cross-seeding and studied the physical–chemical properties of the resulting aggregates, which allowed us to explain the causal factors underlying differences in cytotoxicity of Aβ40 and Aβ42 fibrils.

## 2. Results

### 2.1. Aβ40 Amyloids Are Predominantly Individual Thin Fibers, While Aβ42 Amyloids Are Fibrillar Clusters (Agglomerates)

**Figure 1:**
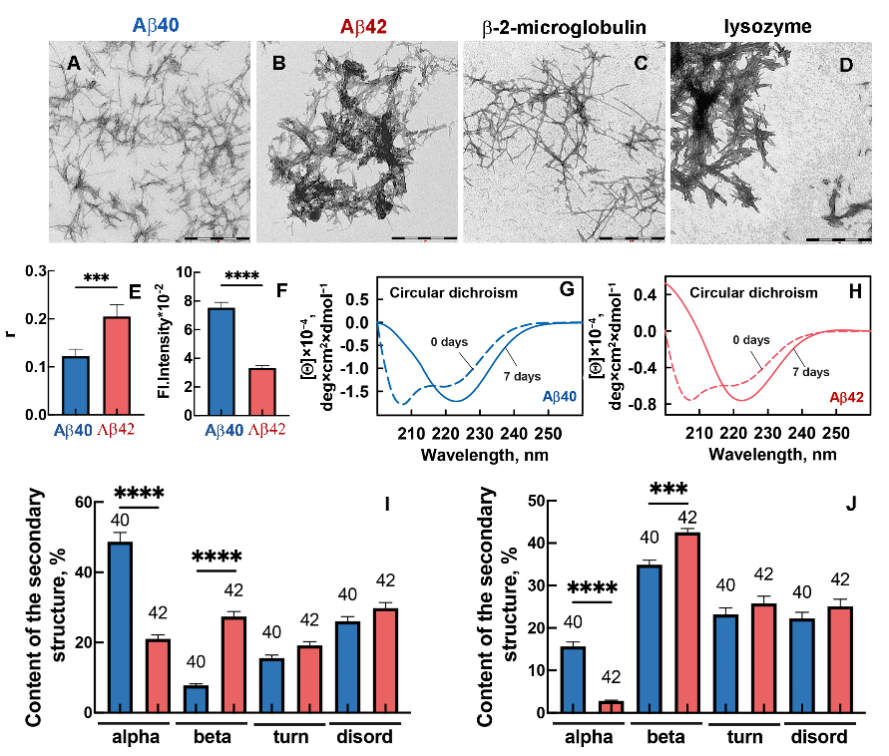
Morphology, structure, and photophysical properties of amyloid fibrils formed from Aβ40 and Aβ42. Transmission electron microscopy images of amyloid fibrils formed from (**A**) Aβ40, (**B**) Aβ42, (**C**) β-2-microglobulin, and (**D**) lysozyme. The scale bar is equal to 1 μm. (**E**) Fluorescence anisotropy (r) and (**F**) fluorescence intensity in the spectrum maximum of Aβ40 and Aβ42 amyloid fibrils. (**G**,**H**) Circular dichroism spectra in the samples with (**G**) Aβ40 and (**H**) Aβ42 before (dashed lines) and 7 days after (straight lines) HFIP evaporation. (**I**,**J**) Content of elements of the secondary structure in the samples with Aβ40 and Aβ42 before (**I**) and 7 days after (**J**) HFIP evaporation. Figure shows content of alpha-helixes (alpha), beta-sheets (beta), beta-turns (turn), and disordered structure (disord). *** *p* ≤ 0.001, **** *p* ≤ 0.0001.

Morphology visualization of the Abeta-peptide amyloids by transmission electron microscopy (Figure 1A,B) showed the long thin fibers, which in the case of Aβ42 had a high tendency to form clusters (Figure 1B). Almost all Aβ42 fibrils were conglomerated in clots, unevenly distributed over the surface of the EM grid.

Individual thin Aβ40 fibers aggregated to a much lesser extent (Figure 1A), and they were more equally spaced across the surface of the EM grid. The larger size of Aβ42 compared to Aβ40 amyloids is confirmed by the data on their fluorescence anisotropy values (Figure 1E). The lower value of intrinsic UV fluorescence of Aβ42 fibrils compared to Aβ40 fibrils, which may result from tyrosine residues entering fibrillar clusters, also supports this observation. It should be noted that amyloid fibrils formed from other amyloidogenic proteins have a similar distinct propensity for agglomeration [33,34]. In particular, Figure 1C,D show individual fibers of beta-2-microglobulin (as in the case of Aβ40) and large fibrillar clots of lysozyme (as in the case of Aβ42). We can assume that the degree of fibril agglomeration may result from (a) the conditions of fibril growth and (b) the fibril spatial structure determined by the structure of individual amyloid-forming monomers. Since we have used identical conditions for amyloid preparation, whereas the amino acid sequence of peptides differs by two residues, we investigated the secondary structure of Aβ40 and Aβ42 peptides forming the fibers.

### 2.2. The Amino Acid Sequence of Abeta Peptides Affects the Secondary Structure of Their Amyloid Fibrils

Circular dichroism (CD) spectra in the far UV region indicated a significant change in the secondary structure of both peptides during fibrillogenesis (Figure 1G,H). For each of the samples, the minimum in the spectral region of 220–230 nm became more pronounced during fibrillogenesis, which displayed an increase in the beta-structural content necessary for the formation of the amyloid fibril backbone. We estimated the content of different types of the secondary structure in the samples with the CDPro program using three regression methods and several basis sets containing soluble, membrane, and denatured proteins (37–56 units in each) with a known secondary structure. The quantitative analysis proved a significant increase in the content of beta-sheets during the formation of Aβ40 and Aβ42 amyloid fibrils (Figure 1I,J show the content of elements of the secondary structure in the samples before and 7 days after HFIP evaporation). These values reached 36% and 43% for Aβ40 and Aβ42, respectively. To compare the structural polymorphism of Aβ40 and Aβ42 peptide chain regions forming the core of the amyloid fibril (beta-folded areas), we studied the interaction of the samples with fluorescent probe thioflavin T (ThT).

### 2.3. There Are Two Types (Modes) of Binding of the Fluorescent Probe Thioflavin T to Aβ42 Amyloids and Just One Type of Binding to Aβ40 Fibrils

The amyloid-specific dye thioflavin T (ThT) has long been used as a test for the formation of amyloid fibrils [29,30,31,32]. Recent studies indicate that ThT can also be a tool for studying the structural polymorphism of amyloids since the parameters of its binding to various fibrils and the photophysical characteristics of the bound dye can differ significantly [33,34,35,36]. The difficulty in studying the interaction of ThT with amyloid fibrils lies in the fact that a sample represents an equilibrium system of free and fibril-bound dye, the photophysical characteristics of which differ significantly. However, the photophysical characteristics of each of the dye fractions are essential to determine the parameters of ThT binding to fibrils. Traditionally, having no way to distinguish the characteristics of the free and fibril-bound dye in a sample, the researchers used the total concentration of ThT to calculate dye–fibril binding parameters, but it is incorrect.

**Figure 2:**
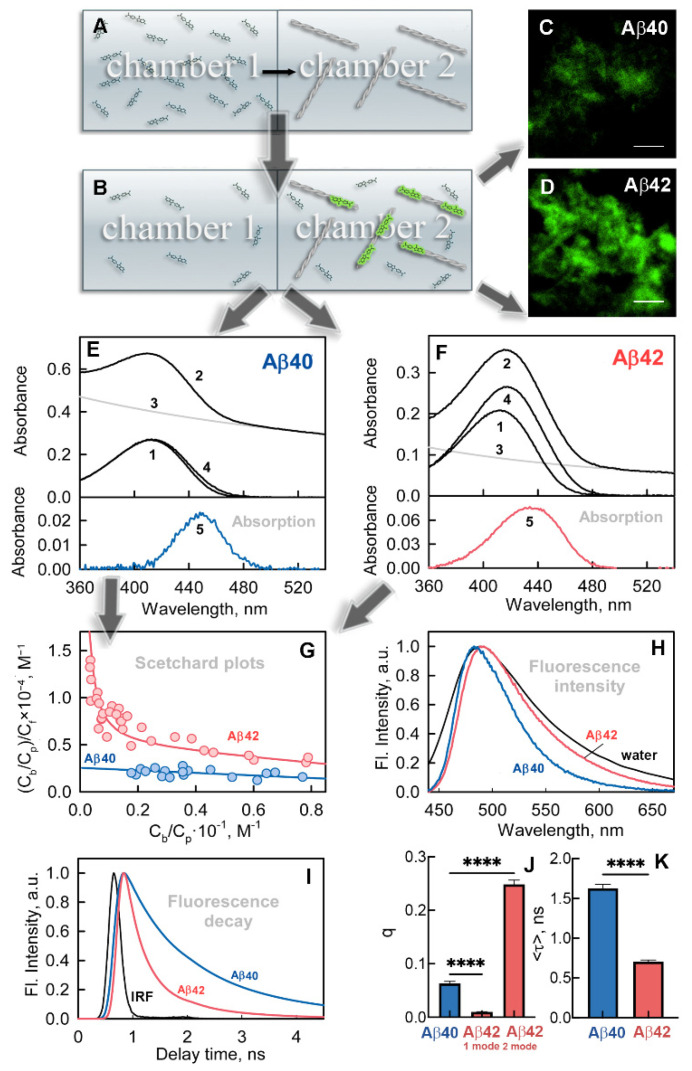
Interaction of Aβ40 and Aβ42 amyloid fibrils with fluorescent probe thioflavin T (ThT). (**A**) Preparation of ThT-fibril test solutions by equilibrium microdialysis. The panel shows two chambers with equal volume (chambers 1 and 2) that are separated by a membrane permeable to dye and impermeable to fibrils. ThT solution was placed in chamber 1; amyloid fibrils were placed in chamber 2. (**B**) Samples after equilibration. The panel shows that free dye concentrations in chambers 1 and 2 become equal after equilibration. At the same time, the total ThT concentration in chamber 2 exceeds that in chamber 1 by the concentration of the bound dye. (**C**,**D**) Confocal microscopy of ThT-stained amyloids formed from (**C**) Aβ40 and (**D**) Aβ42. The scale bar on the images is equal to 4 μm. (**E**,**F**) Determination of the absorption spectra of ThT bound to amyloid fibrils from (**E**) Aβ40 and (**F**) Aβ42. Curves 1 and 2 represent absorption spectra of ThT in chamber 1 (free ThT) and chamber 2 (superposition of absorption spectra of free ThT, ThT bound to fibrils, and apparent absorption caused by fibril light scattering) after equilibration. Curve 3 is the optical density determined by fibril light scattering. Curve 4 is the total absorption of free and bound ThT after light scattering subtraction. Curve 5 is the absorption spectra of ThT bound to amyloid fibrils. (**G**) Scatchard plots characterizing ThT binding to amyloid fibrils formed from Aβ40 (blue) and Aβ42 (red). The dots in the Figure show the experimental values. The approximating curves for experimental data were calculated by SigmaPlot software. (**H**) Normalized fluorescence spectra of ThT in a free state (black) and bound to amyloid fibrils formed from Aβ40 (blue) and Aβ42 (red). (**I**) Fluorescence decay curves of ThT bound to amyloid fibrils formed from Aβ40 (blue) and Aβ42 (red). The figure also shows the instrument response function (IRF). (**J**,**K**) The values of the (**J**) fluorescence quantum yield and (**K**) lifetime of the dye bound to fibrils. **** *p* ≤ 0.0001. Arrows used on the figure show the sequence of experiments and calculations.

To differentiate between characteristics of free and fibril-bound dye, we used a previously developed custom-designed approach. The unique feature of this approach is the preparation of the test samples by equilibrium microdialysis [27,37]. We loaded one of the chambers of the device for equilibrium microdialysis with the dye and loaded the other with fibrils (Figure 2A) and then left the system to equilibrate. After equilibration (Figure 2B), in the first chamber, we obtained a reference solution containing only free ThT molecules. In the second chamber, we obtained a sample solution containing both free ThT at the same concentration as in the reference solution together with a fibril-bound dye. In this case, the absorption and fluorescence spectra of the bound dye can be determined as the difference spectra of the sample and reference solutions. After equilibrium microdialysis, we analyzed the sample solution using fluorescence confocal microscopy, which confirmed the different sizes of the agglomerates formed by Abeta peptides (Figure 2C,D). Then, the absorption spectra of the reference solution (Figure 2E,F, curve 1) and the sample solution (Figure 2E,F, curve2) were recorded. These spectra allowed us to determine the absorption spectra of the dye bound to the fibrils (Figure 2E,F, curve 5). Fibril light scattering (Figure 2E,F, curve 3) was taken into account using the standard protocol [38]. The significant shift of the absorption spectra of fibril-bound ThT (Figure 2E,F, curve 5) to longer wavelengths compared to the absorption spectra of a free dye (Figure 2E,F, curve 1) was shown for both types of studied fibrils. This shift can be ascribed to a change in the microenvironment of dye molecules interacting with amyloids. At the same time, the absorption spectra of ThT bound to Aβ40 and Aβ42 fibrils differ markedly in shape and position of the maximum, confirming the polymorphism of the studied aggregates (Figure 2E,F, curve 5). In particular, the absorption spectrum of ThT bound to Aβ42 fibrils is broader and has a higher intensity than that of ThT bound to Aβ40 fibrils. The concentrations of free dye (C_f_) and the concentration of dye bound to amyloid fibrils (C_b_) were determined from the recorded absorption spectra of ThT.

Equilibrium microdialysis for each type of fibrils was performed multiple times using different concentrations of the dye loaded into chamber 1 while keeping the concentration of fibrils (C_p_) loaded into chamber 2 constant. The Scatchard curves were plotted (Figure 2G), based on a set of obtained C_f_ and C_b_ values. Linearity of the Scatchard plot characterizing the binding of ThT to Aβ40 fibrils suggests the occurrence of the single binding type (mode). The graph characterizing the binding of the dye to Aβ42 fibrils is nonlinear. This gives us evidence of the existence of at least two modes of the dye binding to fibrils having not only different values of the binding constant but also different numbers of binding sites per protein molecule. This assumption is in good agreement with the broadening of the recorded absorption spectrum of ThT bound to Aβ42 fibrils as compared to that of ThT bound to Aβ40 fibrils. The reason for this may lie in the superposition of absorption spectra of dye molecules associated with different modes.

The values of binding constants and the number of ThT binding sites were determined by fitting the experimental data with the method of multiple nonlinear regression (see [39] for detailed calculation protocol) considering the existence of one and two binding modes in the case of Aβ40 and Aβ42 fibrils, respectively. The calculated values of the binding parameters are shown in Table 1. As the calculated values of binding parameters give a satisfactory approximation of the experimental data, the validity of the former, as well as the correctness of the assumed models of the dye interaction with fibrils, can be considered. It should be noted that both Aβ40 and Aβ42 fibrils have one ThT binding mode characterized by similar values of binding constants, assuming a common mechanism of dye interaction. Compared to the first binding mode, the second binding mode of the dye to Aβ42 fibrils is characterized by a 2-order-higher binding constant and a 2-order-lower number of binding sites.

Fluorescence spectra (Figure 2H) and fluorescence decay curves (Figure 2I) of the fibril-bound dye were also recorded using solutions prepared by equilibrium microdialysis. It was shown that the fluorescence spectra of ThT bound to Aβ40 and Aβ42 fibrils, as well as absorption spectra, differed significantly in shape. The fluorescence spectrum of ThT bound to Aβ42 fibrils has an additional shoulder, which is probably associated with the existence of the second dye binding mode.

Availability of the recorded fluorescence spectra of the samples, corrected for the primary inner filter effect, as well as the calculated concentrations of the bound dye, allowed us to estimate the fluorescence quantum yield of ThT bound to Abeta-peptide amyloid fibrils (Figure 2J) (for a detailed calculation protocol, see [42]). In addition, the fluorescence lifetimes of the fibril-bound dye were determined (Figure 2K) using recorded fluorescence decay curves (Figure 2I). It was shown that the calculated values of the fluorescence quantum yield and lifetime of ThT bound to amyloid fibrils significantly exceed the value of these parameters for a free dye in an aqueous solution (Table 1). The reason for a low value of the fluorescence quantum yield and the lifetime of free ThT is in its intrinsic property to behave as a molecular rotor: in the excited state, its benzothiazole and aminobenzene rings can rotate around the covalent bond connecting them. When the angle between its planar fragments reaches a value close to 90°, a nonradiative transition to the ground state occurs. A significant increase in the fluorescence quantum yield and lifetime of the dye upon binding to Abeta-peptide amyloid fibrils is associated with restriction of the rotational motion of fragments of the dye in the excited state, caused by the rigidity of the interaction with its fibrillar microenvironment [43]. This, in turn, limits the conformational space of ThT in the excited state. It should be noted that the fluorescence quantum yield of ThT bound with a single mode of Aβ40 amyloid fibrils and with two modes of Aβ42 fibrils increased to a different extent. The same dynamic was observed for the ThT fluorescence lifetime, where for Aβ42 fibrils the total value originating from two modes was calculated. There may be two possibilities explaining the difference in the obtained fluorescence parameters, both of which are associated with a ThT microenvironment within Aβ40 and Aβ42 fibrils: (1) in the excited state of the dye, the rotational mobility of its fragments is restricted to various extents; (2) in the ground state, ThT molecules have different conformations. Identification and analysis of potential binding sites of ThT molecules on Aβ40 and Aβ42 amyloid fibril fragments were carried out by molecular docking and molecular dynamics simulations.

### 2.4. Amyloid Fibrils Formed from Aβ40 and Aβ42 Have Specific ThT Binding Sites Located at the Junction of Adjacent Peptide Subunits

**Figure 3:**
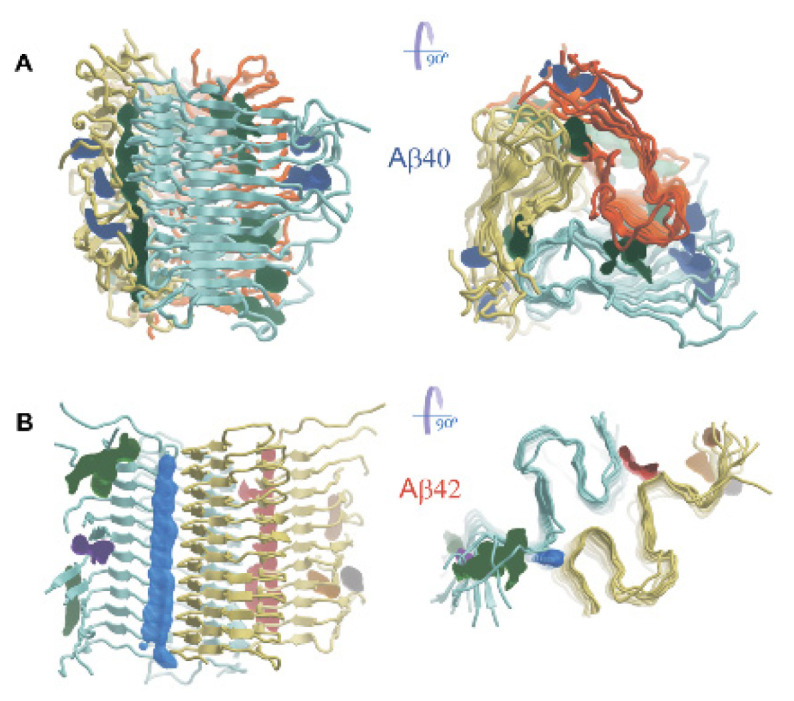
Spatial structure of models of Aβ40 (**A**) and Aβ42 (**B**) fibril fragments, obtained by molecular dynamics simulations. Side (from the left) and top (from the right) views are shown. The filaments forming the fibrils are colored blue-green, yellow, and orange. The volumes enclosed by surfaces indicate potential binding sites for small molecules.

A full-atom model of the Aβ40 fibril fragment was constructed using the spatial structure obtained by solid-state NMR and electron microscopy (PDB code 2M4J) and previously published in [25]. The amyloid-containing material that served as a source of seeds for the growth of fibril, whose structure was deposited in the Protein Data Bank, was extracted from the brain tissue of an Alzheimer’s disease patient. The structure 2M4J is a nonamer composed of the Aβ40 peptide and has third-order symmetry about the fibril axis. In each filament, the PDB entry reports three intermolecular parallel beta layers, involving only short fragments of the polypeptide chain V12–H13 (β1), V18–F19 (β2), and M35–V36 (β3). We constructed a fibril fragment that includes 36 Aβ40 peptide subunits (12 layers of three subunits each) by copying the deposited fragment, consisting of 12 subunits, and translating it along the fibril axis. The stability of constructed fragment was tested by the molecular dynamics simulations (Figure 3A).

The length of the fibril fragment, measured between the Cα atoms of identical amino acid residues located in the terminal strands of the beta layer, was about 55 Å. The diameter, measured as the distance between the Cα atoms belonging to the first residues (N-terminus) of the subunits of one layer, was about 75 Å. Visual analysis showed that in comparison with the idealized model deposited in the PDB (2M4J), where the beta-sheets are planar, the expected twisting of the beta-sheets around the fibril axis is observed as a result of MD simulations (Figure 3A). This twisting is explained by the Ramachandran plot. In a real secondary structure, the energetically favorable values for the dihedral angles of the protein backbone forming individual beta-strands are shifted above the diagonal line, on which the values characterizing the plane beta-sheet are located. In addition, in several subunits, the length of the beta-strand regions ranges entire interval from 10 to 20 amino acid residues, not only the amino acid residues 12–13 and 18–19 as in the case of the 2M4J structure (Figure 3A). Twisting of beta-sheets and the whole fibril fragment leads to disjoining of some adjacent beta-strands and local rupture of two beta-sheets. One of three filaments, retaining the continuous structure of the beta-sheet, partially loses contact between its residues D7-G9 and the residues G25-N27 of the adjacent filament. This results in the formation of a cleft at the junction of the two filaments. The most mobile regions of peptides are the N-terminal sites spanning the first 10 amino acid residues located on the rim of the fibril. The loops formed by residues 22–28 and C-terminal regions covering residues 36–40 are less mobile. In general, the constructed fibril fragment keeps its quaternary structure over 100 ns MD simulations. However, the terminal subunits of the fibril fragment tend to partially lose the beta-structure.

A full-atom model of the Aβ42 fibril fragment was constructed using the spatial structure solved by combining data from solid-state NMR spectroscopy and mass-per-length measurements from electron microscopy images (PDB code 2NAO [26]). The authors have run fibrillogenesis using seed from the recombinant Aβ42 peptide. The spatial structure of the fibril fragment is represented by the Aβ42 peptide hexamer. A single peptide molecule assumes a shape of a double horseshoe-like cross–β-sheet. The protofilament of the fibril contains five intermolecular parallel β-layers comprising residues 2–6 (β1), 15–18 (β2), 26–28 (β3), 30–32 (β4), and 39–42 (β5) of Aβ42 peptides. We constructed a fibril fragment that includes 24 Aβ42 subunits (12 layers of two subunits in each) by copying the deposited fragment consisting of 8 subunits and translating it along the fibril axis.

Figure 3B presents the results of MD simulations demonstrating the stability of the constructed fibril fragment. The length of the fibril fragment, measured between the Cα atoms of identical amino acid residues located in the terminal strands of the beta layer, again, was about 55 Å. The diameter, measured as the distance between the Cα atoms belonging to the first residues (N-terminus) of the subunits of the same layer, was about 90 Å. It can be noted that the double horseshoe-like backbone of the fibril remains reasonably stable. The length of the strands in the β2 and β5 beta layers can increase by one residue. The N-terminal regions spanning residues D1-S8 have the greatest mobility, resulting in losses of the beta-layer β1 secondary structure; only short intermolecular beta-structures comprising 2–3 subunits remain in this region of the polypeptide chain. Loops A21-G29, connecting the beta-layers, as well as the C-terminal regions V39-A42, demonstrate increased mobility. In both Aβ40 and Aβ42 fibril fragment models, the terminal peptide subunits undergo greater conformational changes during MD simulations in comparison with the rest of the enclosed subunits.

**Figure 4:**
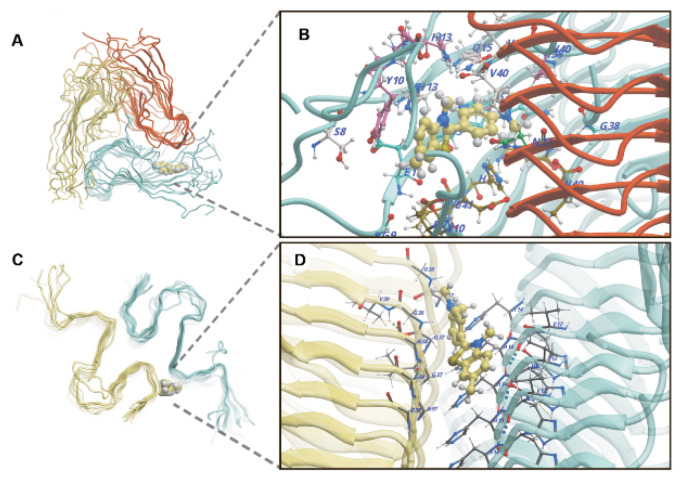
ThT binding sites of Aβ40 (**A**,**B**) and Aβ42 (**C**,**D**) fibrils are characterized by the lowest binding free energy calculated from molecular dynamics simulations; the ThT molecule is displayed with van der Waals spheres. Different colors in the panels (**A**,**B**) show different filaments that form the amyloid fiber. Amino acid residues of peptides interacting with ThT (displayed with ball-and-stick model) at the sites are shown in panels (**B**,**D**) for Aβ40 and Aβ42 fibrils, respectively.

Potential binding sites for small molecules were found using the median structure of Aβ40 and Aβ42 fibrils fragments from the most populated cluster obtained from the analysis of MD trajectories (Figure 4). For the Aβ40 fibril, such sites are located: (1) at the junction of two filaments and are formed by amino acid residues N27-A30 of one filament subunit and G9-V12 and G37-V40 of the adjacent filament subunits (2) between the N-terminal regions of the polypeptide chains, including the first ten amino acid residues. For the Aβ42 fibril potential binding sites are formed: (1) in the junction of two filaments formed by amino acid residues V12-H15 of one filament and G37-G38 of the second filament (2) between the N-terminal regions, including the first ten amino acid residues of the polypeptide chains forming one filament.

In the binding sites identified for the models of Aβ40 and Aβ42 fibril fragments, possible binding modes of the ThT were investigated by molecular docking, taking into account the flexibility of amino acid side chains. Selected representative ThT poses served as starting points for molecular dynamics simulations to test the stability of dye binding at these sites. For the Aβ40 fibril, the lowest value of binding free energy (−42.7 ± 4.2 kcal/mol) corresponds to the poses of ThT in a closed cavity formed at the junction of two filaments (Figure 4A,B). It can be assumed that at the moment when the N-terminus of one filament comes away from the G37-V40 region of the adjacent filament, ThT can intrude into such a site. Inside the cavity, ThT can marginally rotate, but motion along the main axis of the filament is constrained. For ThT located at this site, the dihedral angle distribution between the aminobenzene and benzothiazole rings has two maxima at 40° and 130° (Figure S1A), while free ThT in an aqueous environment has maxima at 55° and 125° (Figure S1C). In the Aβ42 fibril, the highest ThT binding affinity is observed at the external junction region between two filaments. However, here ThT can freely move within the groove extending along the main axis of the filament. As with the Aβ40 fibril, for the Aβ42 fibril, the dihedral angle distribution between the aromatic rings also has two maxima around 40° and 130° (Figure S1B). The minimum binding free energy of ThT is −37.1 ± 5.6 kcal/mol. As noted earlier, in addition to the described binding sites, ThT can nonspecifically interact with the N-terminal residues of both types of fibrils with minimum binding free energies of −32.5 ± 4.1 kcal/mol and −28.8 ± 3.8 kcal/mol for Aβ40 and Aβ42, respectively.

Based on extensive molecular dynamics simulation, we have found new sites for stable ThT binding in the junction of Aβ40 and Aβ42 fibril filaments, which were not reported previously by Peccati et al. [44]. We believe that the penetration of the dye into the binding sites located deep inside the fibril is possible only when the dye is added during its growth. At the same time, during staining of the mature fibril, dye possibly can incorporate only into sites located either directly on the surface or close to the surface, periodically becoming accessible due to the mobility of the terminal fragments of the peptides forming the fibril. The MM/GBSA binding free energy of ThT with the Aβ40 fibril calculated here has a close value to that published previously (−42.7 vs. −37.6 kcal/mol). At the same time, the external-junction site at the surface of Aβ42 fibrils is characterized by the significantly lower binding free energy of ThT (−37.1 vs. 15.0 kcal/mol); i.e., binding to this site is more likely. Frieg et al. [45] investigated the binding of ThT to a model of another Aβ42 fibril polymorph, built based on the structure recorded in the 5OQV PDB entry. The authors do not describe the binding of ThT at the junction of the two filaments. The sites they found on the fibril surface are characterized by higher binding free energies.

Despite the revealed topological differences in the binding sites of the dye and its orientation relative to the axis of the Aβ40 and Aβ42 fibrils, in general, the mechanism of ThT interaction with these amyloid fibrils is similar: the ThT molecule is incorporated into the interfaces between adjacent peptide subunits located in two filaments. Thus, within the approximation of the presented model obtained using computational approaches, we have characterized the single mode of ThT binding to Aβ40 and Aβ42 amyloid fibrils.

### 2.5. The Second Mode of ThT Binding to Aβ42 Amyloids Probably Originates from the Localization of the Dye Molecules in the Areas of Fibril Clustering

The literature data analysis revealed that the ThT binding mode to Aβ40 and Aβ42 fibrils characterized by the affinity of the order of 10^−4^ M was reported previously for amyloids formed from other amyloidogenic proteins (Table 1). The presence of this binding mode confirms the similarity of the general morphology of investigated amyloid fibrils and the identity of the way ThT binds with them (according to our results, the dye is incorporated into the junction between adjacent protein and peptide subunits that form the fibril backbone). It can be noted that the number of binding sites for this mode varies nearly 10-fold (Table 1), which can be associated with the accessibility of these sites and the specificity of their location on the fibril fiber. Another characteristic of discussed binding mode that can vary significantly (for example, in the case of lysozyme and Abeta peptide, see Table 1) is the ThT binding constant. This constant may depend both on the heterogeneity of amino acid residues forming the binding sites and on the specific structural features of the amyloid fiber (the number of protofilaments, the packing density of protofibrils in the fibril, its flexibility, etc.). However, such differences can hardly explain an increase in the affinity of ThT interaction with fibrils by 2 orders of magnitude (up to 10^−6^ M^−1^) and a decrease in the number of binding sites by almost 2 orders of magnitude, as is observed in the case of the second binding mode to fibrils formed from Aβ42 or other amyloidogenic proteins (Table 1). Earlier, we assumed that this ThT binding mode is associated with the incorporation of the dye into the amyloid clots [33]. In this case, the dye molecule can colocalize in binding sites specific to the first mode, but its mobility can be additionally restricted by the adjacent amyloid fiber. This idea is supported by electron microscopy images, showing that fibrils formed from Aβ42, insulin, and lysozyme, which have a secondary ThT binding mode, actually tend to aggregate and form clots (Figure 1B,D). In contrast, Aβ40 and beta-2-microglobulin amyloid fibrils, for which a single ThT binding mode was observed, appear as separate thin filaments (Figure 1A,C). The results obtained suggest that the number of ThT binding modes to amyloid fibrils, as well as the number of high-affinity binding sites within this mode, can be predictive of the degree of amyloid fibril clustering.

### 2.6. Abeta-Peptide Amyloid Fibrils with a Greater Tendency to Form Clusters Exhibit Stronger Resistance to Destabilizing Effects

Previously we reported the hypothesis about the interplay between the degree of amyloid fibril clustering and the stability of amyloid fibrils [46]. Here, this hypothesis is further proved by the example of Abeta-peptide amyloid fibrils.

**Figure 5:**
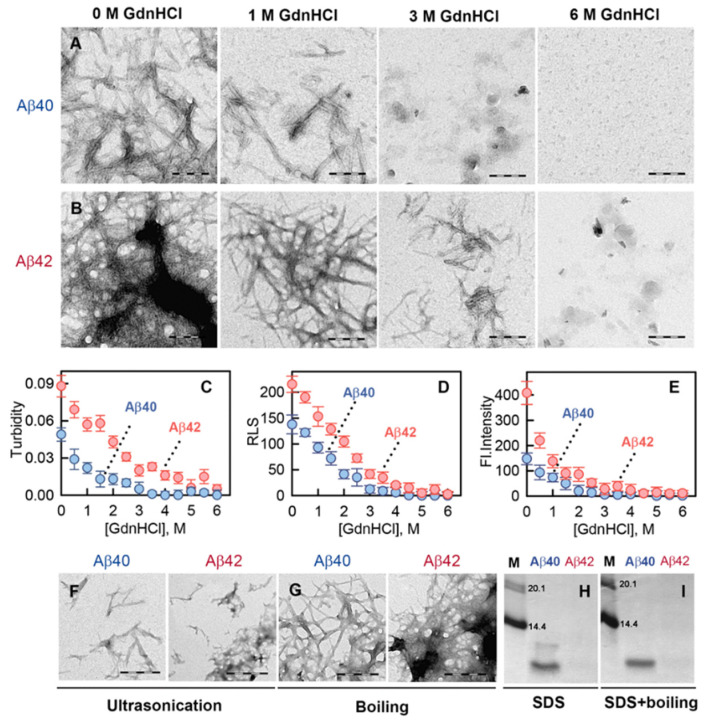
Stability of amyloid fibrils formed from Aβ40 and Aβ42. (**A**,**B**) Transmission electron microscopy of Aβ40 (**A**) and Aβ42 (**B**) fibril degradation induced by GdnHCl. Denaturant concentration is indicated above the panels. The scale bar on the images is equal to 250 nm. Change in (**C**) turbidity, (**D**) Rayleigh light scattering (RLS), and (**E**) fluorescence intensity of amyloid fibrils formed from Aβ40 (blue) and Aβ42 (red). (**F**,**G**) Transmission electron microscopy of Aβ40 (left panels) and Aβ42 (right panels) fibrils after (**F**) ultrasonication and (**G**) boiling. The scale bar on the images is equal to 300 nm. (**H**,**I**) Detergent resistance of Aβ40 and Aβ42 amyloid fibrils. The figure shows electrophoregrams of (**H**) unboiled samples treated with 2% cold SDS and (**I**) boiled samples treated with 2% SDS. The molecular markers are in the left lanes (bands corresponding to *M*_W_ = 20.1 and 14.4 kDa are shown).

We investigated the effect of denaturing agent guanidine hydrochloride (GdnHCl) on Aβ40 and Aβ42 fibrils. We analyzed samples after one-day incubation in the presence of 0 to 6 M GdnHCl by electron microscopy (Figure 5A,B). The obtained images indicated that GdnHCl induces two separate processes: degradation of fibrillar clots into individual fibers (predominantly for the Aβ42 amyloid sample) and shortening of the fibrils without changing their backbone structure (in both Aβ40 and Aβ42 amyloid samples). It was shown that even in 3 M GdnHCl solution, Aβ40 fibrils were practically not detected, while in the sample with Aβ42 amyloids, thin shortened fibers (without clustering regions) are still present in a noticeable amount. The obtained results indicate that Aβ42 amyloids are more resistant to denaturant than Aβ40 fibrils. As the method of electron microscopy provides only a qualitative description of the general morphology of amyloid fibrils, to quantitatively characterize fibril disassembly, we investigated the intrinsic photophysical characteristics of the pure samples and their mixtures with ThT at various denaturant concentrations.

The turbidity (an apparent absorption caused by the light scattering) and Rayleigh light scattering (RLS) of the samples began to change even at 0.5 M denaturing agent concentration, which indicates a decrease in the number and/or physical size of the studied aggregates (Figure 5C,D). Our data indicated that the disassembly of fibrillar clots and depolymerization of amyloid fibers started even at low GdnHCl concentrations, while at high GdnHCl concentrations (3–3.5 M and 5 M for Aβ40 and Aβ42 fibrils, respectively), large aggregates are not detected in the samples. This is an unexpected result, given the literature data on the extremely high stability of amyloids [47,48,49,50]. However, similar results on fibril stability were obtained in recent work [46], where we showed the degradation of amyloid fibrils formed from beta-2-microglobulin and lysozyme even in the presence of low concentrations of GdnHCl. To detect the effect of gradually increasing concentration of GdnHCl on the fibril degradation process, we registered the change in the fluorescence intensity of ThT bound with the fibril aggregates under investigation.

For this aim, initially, an evaluation of the GdnHCl effect on the dye fluorescence in the absence of amyloid fibrils was made. Noticeably, an increase in the denaturant concentration to 6 M led to an increase in the dye fluorescence intensity by almost 3 times (Figure S2). Thus, since the ThT solution with fibrils is an equilibrium system of free and fibril-bound dye, an increase in the GdnHCl denaturant concentration in the sample will lead not only to a decrease in the fluorescence intensity of the bound dye due to fibril depolymerization but also to an increase in the fluorescence intensity of the free dye. This means that the correct investigation of amyloid fibril degradation by GdnHCl assumes the proper consideration of the change in the fluorescence intensity of each of the dye fractions obtained using the sample and reference solutions prepared by equilibrium microdialysis. Using these solutions, fluorescence spectra of ThT bound to amyloid fibrils were determined at various concentrations of GdnHCl.

Analysis of the obtained results showed that with an increase in the concentration of the denaturing agent, the fluorescence intensity of the dye bound to the fibrils decreases. At 3 M GdnHCl for Aβ40 fibrils and 4.5 M GdnHCl for Aβ42 fibrils, this value became close to 0 (Figure 5E). This is in good agreement with the electron microscopy data, as well as with the turbidity and RLS values of the samples (Figure 5A–D) indicating fibril degradation. A decrease in the fluorescence intensity of the bound dye already at 0.5 M GdnHCl concentration confirmed the depolymerization of amyloid fibrils even at low concentrations of the denaturing agent. Interestingly, in the Aβ42 sample at GdnHCl concentrations lower than 1 M, a dramatic decay of the ThT fluorescence intensity was registered. It could be ascribed to the degradation of fibrillar clots and elimination of ThT binding sites associated with them, where the dye had a high fluorescence quantum yield. Thus, our results, firstly, indicate that the conventional literature conception of the extreme stability of amyloid fibrils should be revised; secondly, they confirm our assumption about significant differences in the stability of amyloid fibrils formed from Aβ40 and Aβ42. Aβ42 amyloid fibrils are more resistant to destabilizing effects, which may be associated with the peculiarity of the packing of peptides in amyloid fiber or with the ability of these fibrils to form stable clots.

To further prove the distinctions in the stability of Aβ40 and Aβ42 amyloid fibrils, their response to ultrasonication, boiling, and treatment by ionic detergent sodium dodecyl sulfate (SDS) was also investigated. Ultrasonication led to fragmentation of amyloid fibrils with more pronounced lesions in the case of Aβ40 fibrils (Figure 5F). The boiling of Aβ40 and Aβ42 amyloid fibrils did not significantly affect their morphology (Figure 5G). However, the fibrils displayed a distinct response to SDS. The electrophoretic profile of Aβ40 and Aβ42 amyloid samples treated with SDS (to 2% final concentration) is shown in Figure 5H. In the case of Aβ40 fibrils, two bands on the electrophoregram correspond to the monomeric and oligomeric forms of the peptide; in the case of Aβ42 fibrils, no bands on the gel were found, implying Aβ42 amyloids practically did not degrade and, thus, did not enter the gel. After boiling the samples in the presence of SDS, we observed a single well-defined band corresponding to monomeric Aβ40 peptide and did not observe bands corresponding to monomeric Aβ42 peptide (Figure 5I). Results of this experiment prove that Aβ42 amyloids form more detergent-resistant aggregates than Aβ40.

In summary of the described results, for Aβ40 and Aβ42 fibrils, we revealed significant differences not only in the secondary structure, clustering tendency, and interaction with a fluorescent probe but also in their resistance to destabilizing effects. Next, we tested whether these differences affect the cytotoxicity of Abeta-peptide amyloid fibrils.

### 2.7. The Structure and Properties of Abeta-Peptide Amyloid Fibrils Affect Their Cytotoxicity In Vitro

The fibrillar form of Aβ-peptide affects cell viability and metabolism in many ways by binding to cell membranes, shifting protein homeostasis, and facilitating de novo formation of toxic oligomers [51,52]. We estimated whether the observed differences in the structure of Aβ40 and Aβ42 amyloid fibrils affected the toxicity of amyloid fibrils in vitro.

For this aim, we evaluated the effect of the Aβ42 and Aβ40 fibrils on cell viability by MTT assay, which uses the correlation between the metabolic activity of cells and the amount of formazan reduced from 3-(4,5-dimethylthiazol-2-yl)-2.5-diphenyltetrazolium bromide by cellular oxidoreductase enzymes. It should be noted that this cell viability assay has been widely used to investigate amyloid toxicity [53,54], and we wanted to compare our data on fibril-specific toxicity with the results of other studies. This problem is quite current if we take into account the contradictory data on the comparative cytotoxicity of Aβ40 and Aβ42 amyloids. In particular, some authors believe that these fibrils possess comparable intrinsic toxicity [55]; however, others note higher cytotoxicity of Aβ42 amyloids [56]. In addition, in several studies, the authors suggest that Aβ42 amyloids do not affect cell viability at all, and only peptide oligomers are toxic [57].

**Figure 6:**
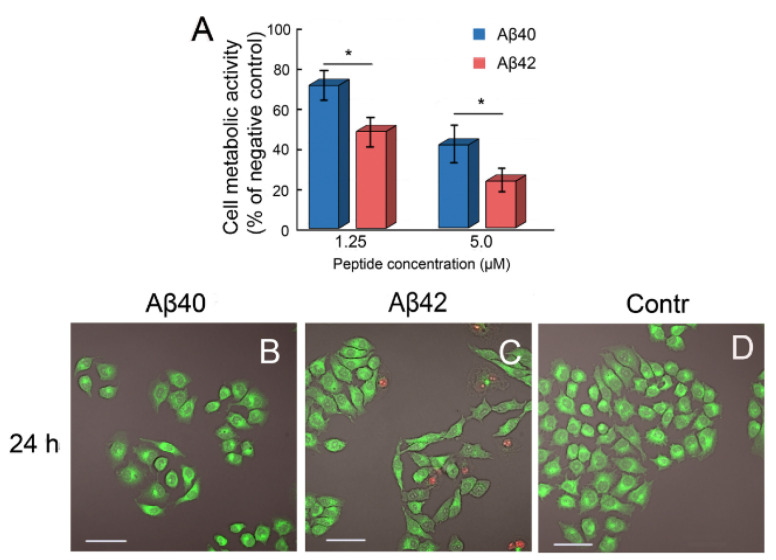
Effects of amyloid fibrils formed from Aβ40 and Aβ42 on HeLa cells. The results of the MTT assay (**A**) and representative photomicrographs taken at 24 h (**B**–**D**) are presented. Experiments were repeated at least 3 times with similar results. ANOVA, Tukey post hoc, * *p* < 0.01. Cells treated with 5 µM of Aβ40 (**B**) and Aβ42 (**C**) amyloids as well as untreated cells (**D**) were imaged with an FV3000 microscope. The scale bar on the images is equal to 50 μm. A green fluorescence is produced by the live cells component. A red fluorescence corresponds to the dead cells with compromised membranes.

In the current study, we demonstrated that both types of fibrils impaired cellular MTT reduction in a dose-dependent manner (Figure 6A), while Aβ42 fibrils resulted in a more pronounced decrease in the metabolic activity of the cells. To elucidate the reasons for the observed decrease in metabolic activity (changes in the level of proliferation or cell death), we estimated the proportion of dead cells in the population using specific fluorescent dyes and phase-contrast microscopy.

Following 24 h of incubation with Aβ40 amyloid fibrils, the cells retained their morphology, and the proportion of dead cells in the population did not exceed 5% (Figure 6B). The proportion of dead cells in the population incubated with Aβ42 amyloids was more pronounced and amounted to 15%. In this population, nuclear condensation and cell elongation were also observed (Figure 6C). It was seen that both types of amyloid fibrils had an antiproliferative effect on HeLa cells, in contrast to the control group, which was characterized by an organized cell monolayer with a regular polygonal appearance (Figure 6D).

In general, the pronounced toxicity of amyloid fibrils we observed is consistent with the results obtained earlier [58] that demonstrated that the fibrillar form of Aβ42 caused the most pronounced changes in cellular metabolism, reflecting significant cell damage. At the same time, we observed neither cell rounding nor granulation, characteristic of apoptotic death, which predominated in neuronal cell cultures upon administration of various oligomeric forms of Abeta peptides [59,60]. However, according to the literature, cells treated with mature lysozyme fibrils showed annexin V/PI double-positive staining, suggesting a late apoptotic or secondary necrotic death [61]. A similar mechanism of cell death can probably be observed under the action of amyloid fibrils formed from Aβ-peptides on the HeLa cell line.

Thus, the observed reduction in metabolic activity, which was more pronounced in the case of treatment by amyloid Aβ42 fibrils, correlated with morphological data. It can be assumed that the clustering tendency of amyloid fibers and their stability are the important factors that, at least in part, may determine the toxicity of amyloid fibrils in vitro.

### 2.8. Cross-Seeding Using Preformed Aβ40 and Aβ42 Amyloids Leads to a Change in the Structure and Properties of Amyloid Fibrils Grown from These Peptides

Taking into account that in the blood plasma and senile plaques of AD patients, in most cases, a mixture of monomeric and aggregated forms of Abeta peptides is detected [62], the study of the structure, properties, and toxicity of Aβ40 and Aβ42 fibrils formed as a result of seeding and cross-seeding is of interest. It was shown that Aβ40 fibril seeds could promote Aβ42 aggregation and vice versa, and the kinetics of this process was characterized [63]. At the same time, by using the sample of amyloid fibrils formed from various amyloidogenic proteins, it was shown that the structure of the amyloid seed could significantly affect not only fibrillogenesis but also the structure of mature amyloids [64]. In this regard, we analyzed the differences in the morphology of Aβ40 and Aβ42 amyloids obtained as a result of seeding and cross-seeding. We denoted seeded fibrils as follows: “Aβ40 and Aβ42 fibrils”—the fibrils grown without seeds; “Aβ40 (Aβ40) and Aβ40 (Aβ42) fibrils”—Aβ40 fibrils prepared in the presence of Aβ40 and Aβ42 amyloid seeds, respectively; “Aβ42 (Aβ40) and Aβ42 (Aβ42) fibrils”—Aβ42 fibrils prepared in the presence of Aβ40 and Aβ42 amyloid seeds, respectively (i.e., the parentheses enclose the name of seed-generating peptide).

**Figure 7:**
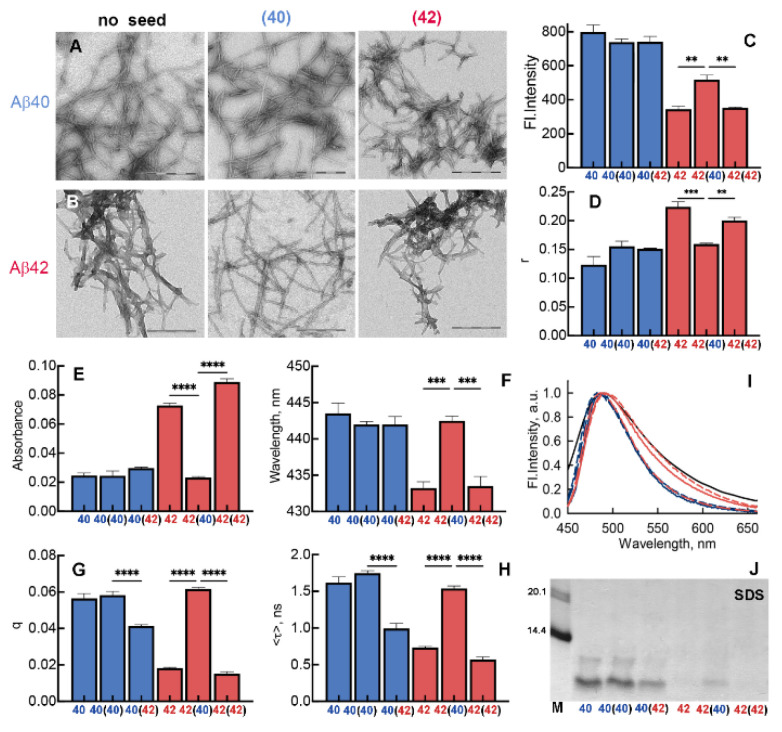
Morphology, structure, and properties of Aβ40 and Aβ42 amyloid fibrils obtained by seeding and cross-seeding. (**A**,**B**) Transmission electron microscopy of (**A**) Aβ40 and (**B**) Aβ42 amyloids obtained from different seeds. The amyloidogenic peptide is indicated on the left of the panels, and the amyloidogenic seed is indicated above the panels. The scale bar on the images is equal to 300 nm. (**C**) Fluorescence intensity and (**D**) fluorescence anisotropy (r) in the spectrum maximum of amyloid fibrils formed from Aβ40 and Aβ42. If a seed is present, it is indicated in the round brackets. (**E**) Absorption in the spectrum maximum, (**F**) wavelength of the absorption maximum, (**G**) fluorescent quantum yield, (**H**) fluorescence lifetime of to fibril-bound ThT, and (**I**) normalized fluorescence spectra. ** *p* ≤ 0.01, *** *p* ≤ 0.001, **** *p* ≤ 0.0001. (**J**) Electrophoregrams of unboiled fibrils treated with 2% cold SDS. The molecular markers are in the left lane (bands corresponding to *M*_W_ = 20.1 and 14.4 kDa are shown).

The Aβ40 (Aβ40) fibrils appeared to be similar in morphology to the Aβ40 fibrils (Figure 7A). At the same time, the addition of an Aβ42 amyloid cross-seeds resulted in the formation of Aβ40 fibril clots. Aβ42 and Aβ42 (Aβ42) amyloid fibrils appeared identical and tended to form clusters (Figure 7B). However, the presence of a cross-seed formed from Aβ40 amyloids significantly altered the morphology of Aβ42 aggregates. Mature Aβ42 (Aβ40) amyloid fibrils were similar in morphology and level of clustering to fibrils Aβ40 and Aβ40 (Aβ40).

The intrinsic fluorescence characteristics of amyloid fibrils obtained as a result of seeding and cross-seeding were investigated. It was shown that the intrinsic UV fluorescence intensity and the fluorescence anisotropy within two groups of fibrils grown from Aβ40 and Aβ42, specifically Aβ40, Aβ40 (Aβ40), and Aβ40 (Aβ42) and Aβ42 and Aβ42 (Aβ42), were similar (Figure 7C,D). At the same time, the fluorescence intensity value of Aβ42 (Aβ40) fibrils was found between the values of Aβ40 and Aβ42 fibrils, and its fluorescence anisotropy was similar to the values for Aβ40 fibrils. Thus, through a comparative analysis of the intrinsic photophysical characteristics of fibrils, we concluded that cross-seeding can transform the properties of Aβ42 amyloid fibrils.

We obtained similar results measuring photophysical characteristics of fibril-bound ThT. We showed that the absorption intensity, the position of the maximum of absorption spectra, the shape of the fluorescence spectra, the value of the fluorescence quantum yield, and the fluorescence lifetime of ThT bound to Aβ42 (Aβ40) amyloid fibrils practically overlapped with these characteristics for Aβ40 and Aβ40 (Aβ40) fibrils, but differed markedly from these characteristics in the case of Aβ42 and Aβ42 (Aβ42) fibrils (Figure 7E–H). Interestingly, the values of fluorescence quantum yield and lifetime of ThT bound to Aβ40 (Aβ42) fibrils were intermediate between the values measured for ThT bound to Aβ40 and Aβ42 amyloids. This may indicate the presence of Aβ40 (Aβ42) fibrils with different morphology in the sample. Thus, the analysis of ThT interaction with amyloid fibrils revealed the effect of cross-seeding on the structure not only of Aβ42 but also of Aβ40 amyloids.

Since we showed different resistance of Aβ40 and Aβ42 amyloid fibrils to SDS treatment, we also analyzed the resistance to this ionic detergent of amyloid fibrils grown by seeding and cross-seeding. It turned out that the use of the Aβ40 seed did not increase the stability of Aβ40 (Aβ40) amyloid fibrils. At the same time, the use of preformed Aβ42 fibrils as a seed led to an increase in the resistance of Aβ40 (Aβ42) fibrils to detergent, since on the electrophoregram the bands corresponding to the monomeric and oligomeric forms of the peptide became less pronounced. No clear differences in the stability of Aβ42 amyloid fibrils grown with and without Aβ42-based seed were found. At the same time, for the Aβ42 (Aβ40) sample, the more pronounced band corresponding to the monomeric peptide was observed. Thus, we have shown the usage of cross-seeding affects not only the structure but also the resistance of Aβ40 and Aβ42 amyloid fibrils to destabilizing effects.

**Figure 8:**
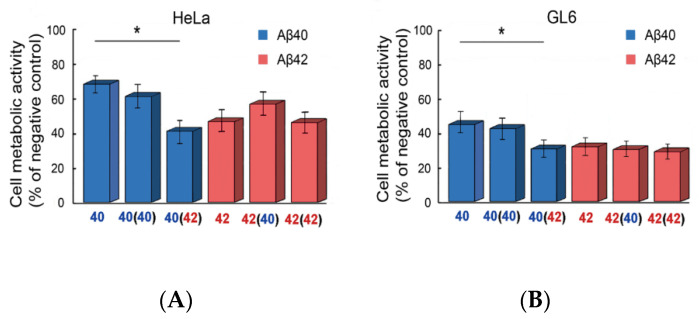
Cytotoxicity of Aβ40 and Aβ42 amyloid fibrils obtained by seeding and cross-seeding. MTT assay of HeLa (**A**) and GL6 (**B**) cell lines treated with 1.25 µM of Aβ40 and Aβ42 amyloids obtained by different seeds. If the seed is used, it is indicated in the round brackets. ANOVA, Tukey post hoc, * *p* < 0.01.

We also analyzed the toxicity of Aβ40- and Aβ42-based amyloids obtained by seeding and cross-seeding for Hela and GL6 cells. According to the MTT assay, all Aβ42 fibril variants had a similar inhibitory effect on cells (Figure 8A). The cytotoxicity of Aβ40 and Aβ40 (Aβ40) fibrils also did not differ. However, cross-seeding changed the inhibitory effect of Aβ40 (Aβ42) amyloid fibrils on both cell lines, increasing it to the level of Aβ42 fibrils. The results obtained allow us to conclude that the presence of an amyloid seed, which affects the structure, level of clustering, and stability of amyloid fibrils, can also alter their cytotoxicity.

## 3. Discussion

**Scheme 1:**
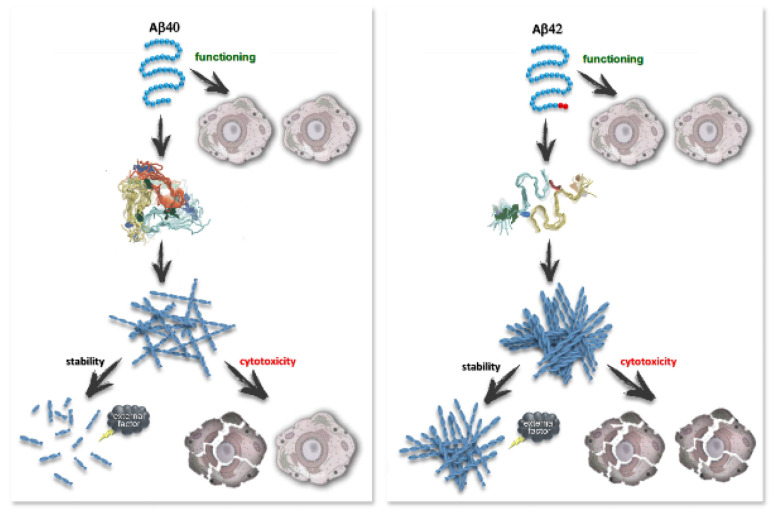
Effect of the primary structure of Abeta peptide on amyloid fibrils formed from them. The scheme shows that the addition of just two C-terminal amino acid residues causes significant differences in the secondary and quaternary structure, aggregation tendency, resistance to destabilizing factors (denaturants, ionic detergents, ultrasonication), and cytotoxicity of amyloid fibrils formed from Aβ40 and Aβ42.

In this work, using a wide range of physicochemical methods, differences in the photophysical properties, secondary structure, and morphology of amyloid fibrils formed from Aβ40 and Aβ42 at the same conditions were shown (Scheme 1). The custom-designed approach based on the preparation of tested samples by equilibrium microdialysis allowed us for the first time to correctly determine and compare the binding parameters of amyloid-specific probe thioflavin T to Aβ40 and Aβ42 amyloid fibers as well as photophysical characteristics of the bound dye. New binding sites of thioflavin T located at the junction between neighboring filaments of fibrils were predicted by molecular modeling techniques. An additional mode of probe interaction with Aβ42 amyloids was experimentally proven, presumably originating from Aβ42 fibril clustering. We have demonstrated that distinct structural features and propensity for clustering of Aβ-peptide fibrils determine specificities not only in their interaction with the fluorescent probe but also in amyloids’ stability (Scheme 1). Aβ42 amyloid fibrils, which form the large clots, are significantly more resistant to destabilizing factors (denaturants, ionic detergents, ultrasonication) compared to unconsolidated individual Aβ40 fibrils, and they have a thinner shape. The availability of the sites that may be affected by external factors is probably one of the factors that determined the amyloids’ stability. This assumption is in good agreement with the literature data [65], indicating that the disintegration activity of serine protease HTRA1 significantly increases the rate of proteolytic degradation of densely packed areas of amyloid fibrils.

**Scheme 2:**
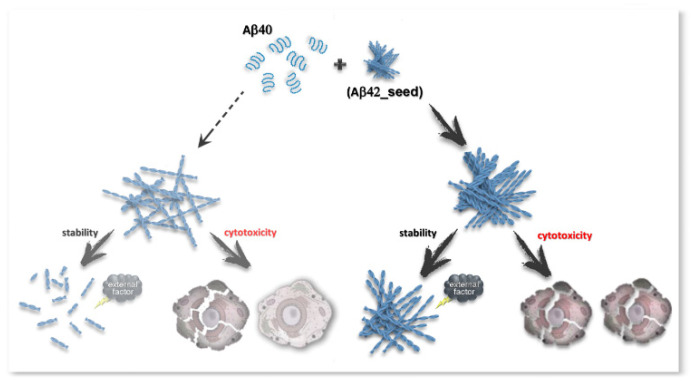
Effect of cross-seeding on Abeta-peptide amyloid fibrils. The scheme shows that fibrillogenesis of Aβ40 in the presence of the previously performed Aβ42 amyloids leads to the predominant formation of fibrils with a higher agglomeration tendency, higher resistance to destabilizing factors, and higher cytotoxicity compared to Aβ40 amyloids formed in the absence of a seed.

We also have demonstrated differences in cytotoxicity of Aβ40 and Aβ42 amyloids (Scheme 1). We assumed that the clustering tendency of amyloid fibers and their stability are the important factors, which, at least in part, may determine the toxicity of amyloid fibrils in vitro. It is believed that the cytotoxic effect of amyloid fibrils is primarily associated with disruption of the cell membranes, where Aβ42 oligomers and fibrils are more toxic than aggregates formed from Aβ40 [66]. By adding preformed fibrils to the cell culture, here we provided support for the direct effect of mature amyloids on the cells resulting in a decrease in their metabolic activity. It must be emphasized that amyloids grown with cross-seeding take on the properties of the seed used. In particular, we have shown that the growth of Aβ40 fibrils by cross-seeding with preformed Aβ42 amyloids caused a cytotoxicity increase to the level of pure Aβ42 amyloids (Scheme 2). A different effect on the metabolic activity of the cells can be due to observed structural features and stability of Aβ40 and Aβ40 (Aβ42). Perhaps this effect is achieved by the formation of additional areas of interaction between fibrils and cell membranes. Interestingly, despite changes in the morphology and level of clustering of Aβ42 (Aβ40) fibrils in comparison to Aβ42 amyloids, it was not possible to restore cellular metabolite activity to the level of Aβ40 amyloids.

In conclusion, in this work, we have clearly shown that the distinction of Aβ-peptides by only two terminal amino acids dramatically affects the overall characteristics of fibrils formed from these peptides: morphology, content of different types of secondary structure, stability, photophysical properties, and interaction with fluorescent probes. The here-identified differences in the clustering tendency and resistance to external factors of Aβ40 and Aβ42 fibrils might be coupled with distinct roles they play in amyloid deposition and consequent distinctions in Alzheimer’s disease pathogenicity in vivo [67].

## 4. Materials and Methods

### 4.1. Materials

Fluorescent dye thioflavin T (ThT) “UltraPure Grade” (AnaSpec, Fremont, CA, USA), isopropyl-beta-D-1-thiogalactopyranoside (IPTG; Fluka, Switzerland), buffer components, and 3-(4,5-dimethylthiazol-2-yl)-2,5-diphenyltetrazolium bromide (MTT) from Sigma (USA) were used without further purification. For amyloid preparation, Aβ40 and Aβ42 peptides from GL Biochem, Shanghai, China, and 1,1,1,3,3,3-hexafluoro-2-propanol (HFIP) from Sigma-Aldrich, St. Louis, MO, USA, were used. DMEM (glucose 4.5 g/L), fetal bovine serum (FBS), 0.25% trypsin-EDTA were purchased from Gibco (Thermo Fisher Scientific, Waltham, MA, USA). Culture flasks and 96-well plates (flat bottom) were purchased from Corning (New York, NY, USA). Dulbecco’s Modified Eagle Medium (DMEM), fetal bovine serum (FBS), penicillin, and streptomycin were purchased from Gibco BRL (Life Technologies, Paisley, Scotland). The human cervical cancer cell line (HeLa) was obtained from the cell bank (The Center of Collective Usage “Collection of Vertebrate Cell Cultures”, Institute of Cytology, RAS)). 3-(4,5-Dimethyl-thiazol-2-yl)-2,5-diphenyltetrazolium bromide (MTT), rhodamine 123, and propidium iodide were purchased from Sigma-Aldrich (St. Louis, MO, USA).

### 4.2. Amyloid Fibril Preparation

Aβ40 and Aβ42 were dissolved in 50% organic solvent 1,1,1,3,3,3-hexafluoro-2-propanol (HFIP, final concentration was 1 mg/mL) and incubated for 7 days [68,69]. The preliminary dissolution of peptides in HFIP allowed us to obtain a unified initial sample that contained no aggregates. HFIP is widely used to provide an entirely monomeric state of proteins and peptides before starting identical aggregation experiments. Moreover, a method was previously developed for the standardized and biocompatible preparation of aggregate-free Abeta peptide for biophysical and biological studies of Alzheimer’s disease [68], which includes the stage of the peptide dissolution in HFIP. Afterward, the HFIP was slowly evaporated under a stream of nitrogen, the volume of the sample was adjusted with distilled water to the initial one, and the samples were stirred for an additional 7 days. These conditions were also used for experiments with seeding and cross-seeding. Pre-prepared fibrils formed from Aβ40 and Aβ42 were used as the seeds. These seeds were added to the samples at the beginning of fibrillogenesis in a 5% (*v/v*) concentration.

For the preparation of amyloids and further experiments, we used a concentration of Aβ of about 0.5–1 mg/mL, which is a standard concentration for peptide fibrillogenesis in vitro [70,71]. It should be kept in mind that the rate of fibril growth significantly depends on the concentration of the peptide. This is the optimal concentration at which the fibril growth time is about 7 days. It should be noted that the concentration of Aβ peptide in brain extracellular fluid is about 10^−10^–10^−9^ M [72,73,74], i.e., several orders of magnitude lower than the concentration at which our in vitro studies were conducted. According to modern concepts, the concentration of the target protein (in our case, the Aβ-peptide) can significantly increase (by several orders of magnitude) due to liquid–liquid phase transitions and the formation of membrane-less organelles also called condensates and bodies [75,76]. The violation of the dynamical structure of membrane-less organelles can lead to the irreversibility of the target protein increase and consequent fibril formation (see, e.g., [77,78,79]).

### 4.3. Transmission Electron Microscopy

Micrographs were obtained using a Libra 120 transmission electron microscope (Carl Zeiss, Oberkochen, Germany). The samples were placed on copper grids coated with formvar/carbon films (Electron Microscopy Sciences, Hatfield, PA, USA). To obtain electron micrographs, the method of negative staining with a 1% aqueous solution of uranyl acetate was used.

### 4.4. ThT-Amyloid Fibril Sample Preparation

The tested ThT-fibril solutions were prepared by equilibrium microdialysis using a Harvard Apparatus/Amika device (Massachusetts, MA, USA). Equilibrium microdialysis was performed with a concentration of aggregates of ~0.5 mg/mL and an initial concentration of ThT of ~32 μM. Spectroscopic study of the sample and reference solutions prepared by the proposed approach allowed us to determine the photophysical characteristics of ThT bound to tested amyloids.

### 4.5. Preparation of the Samples for the Study of Amyloid Stability

For the preparation of the samples of amyloid fibrils with different concentrations of a denaturing agent, an 8 M GdnHCl stock solution was used. The GdnHCl concentration in the tested samples was controlled by refractive index [80] measurement using an IRF-45452M refractometer (Kazan, Russia).

A water bath-type ultrasonic transmitter Elmasonic P30H with a temperature controller (Elma GmbH, Singen, Germany) was used for the decreasing of fibrillary clusters and defragmentation of amyloid fibrils. The volume of the water bath was about 2.5 L. The instrument frequency was 37 kHz, and the power output was set to deliver a maximum of 400 watts. Samples were ultrasonicated from three directions (i.e., two sides and bottom) for 5 min at 37 °C.

For the study of the temperature stability, the samples in concentration 0.5 mg/mL were boiled for 30 min and visualized by TEM. For the study of the stability in response to the effect of ionic detergents, we added SDS (to 2% final concentration) to the samples with Aβ40 and Aβ42 amyloids and loaded them (boiled or not boiled) onto 15% SDS-PAGE gel. A standard method SDS-PAGE [81] was used.

### 4.6. Spectral Measurements

The absorption spectra of the samples were recorded using a U-3900H spectrophotometer (Hitachi, Tokyo, Japan). The absorption spectra of amyloid fibrils and ThT in the presence of the fibrils were analyzed along with the light scattering using a standard procedure [39]. The concentrations of ThT were determined using the molar extinction coefficient of ε412 = 31,600 M^−1^ cm^−1^, and concentrations of fibrils formed from Aβ40 and Aβ42 were determined using the molar extinction coefficient ε280 = 1490 M^−1^ cm^−1^.

CD spectra in the far-UV region were measured using a J-810 spectropolarimeter (Jasco, Tokyo, Japan). Spectra were recorded in a 0.1 cm cell from 260 to 200 nm. For all spectra, an average of three scans was obtained. The CD spectrum of the appropriate buffer was recorded and subtracted from the sample’s spectra. We attempted a quantitative analysis of the secondary structure with the CDPro program [82] using three different regression methods (Selcon, Contin, and CDSSTR) and several basic sets of proteins with a known secondary structure (the sets include from 37 to 56 soluble, membrane, and denatured proteins with different content of the secondary structure).

Fluorescence spectra were measured using a Cary Eclipse spectrofluorometer (Varian, Melbourne, Australia). Fluorescence of the fibrils was excited at 295 nm and registered at 300–450 nm.

The anisotropy of tyrosine fluorescence was calculated using the Equation (1):

(1) $$r=\frac{\left(I_{V}^{V}-GI_{H}^{V}\right)}{\left(I_{V}^{V}+2GI_{H}^{V}\right)}$$

where $I_{V}^{V}$ and $I_{H}^{V}$ are vertical and horizontal components of the fluorescence intensity excited by vertically polarized light, respectively, and $G=I_{V}^{H}/I_{H}^{H}$ is the coefficient that determines the different instrument sensitivity for the vertical and horizontal components of the fluorescence light [83].

The fluorescence of ThT was excited at a wavelength of 440 nm. The spectral slits’ width was 5 nm in most experiments. Changing the slit widths did not influence the experimental results. Recorded fluorescence intensity was corrected on the primary inner filter effect with the use of a previously elaborated approach [28]. A PBS solution of ATTO-425, whose fluorescence and absorption spectra are similar to those of ThT, was taken as a reference for determining the fluorescence quantum yield of ThT bound to fibrils. The fluorescence quantum yield of ATTO-425 was taken as 0.9.

### 4.7. Time-Resolved Fluorescence Measurements

Fluorescence decay curves were recorded with a FluoTime 300 (PicoQuant, Berlin, Germany) spectrometer with an LDH-C-440 laser diode head (*λ*ex = 440 nm). The fluorescence of ThT was registered at *λ*ex = 490 nm. The fluorescence lifetime of ThT bound to studied aggregates was calculated using recorded fluorescence decay curves. For this, the measured emission decays were fit to a multiexponential function using the standard convolute-and-compare nonlinear least-squares procedure [84]. In this method, the convolution of the model exponential function with the instrument response function (IRF) was compared to the experimental data until a satisfactory fit was obtained. The fitting routine was based on the nonlinear least-squares method. Minimization was performed according to Marquardt [85].

### 4.8. Confocal Microscopy

For obtaining the fluorescence images of the ThT-stained amyloid structures, an Olympus FV 3000 confocal laser scanning microscope (Olympus, Tokyo, Japan) was used. We used the oil immersion objective with a 60× magnification, numerical aperture NA 1.42, and laser with an excitation line of 405 nm.

### 4.9. Molecular Modeling

The general plan of structural analysis of complexes of Aβ40 and Aβ42 fibril fragments with ThT by molecular modeling methods is represented by seven main stages: (1) construction of short fibril fragments based on structures located in the Protein Data Bank; (2) relaxation of the obtained models by molecular dynamics simulations; (3) calculation of the protonation state of amino acid residues; (4) productive MD run of fibril fragments and cluster analysis of the trajectory; (5) search for potential binding sites on the surface of fibril fragments and docking of ThT molecules in them; (6) stability testing of complexes of fibril fragments with ThT by MD methods; (7) analysis of the complex’s MD trajectories and calculation of the ThT binding free energy in the obtained binding sites.

Spatial structures with PDB codes 2M4J and 2NAO were selected from the Protein Data Bank to construct atomic models of fragments of Aβ40 and Aβ42 fibrils, respectively. The 3D structure of the Aβ42 amyloid fibrils is composed of two molecules per layer, which are C2-symmetric concerning the central axis of the fibril, while the Aβ42 layer is composed of three molecules and has C3-symmetry to the same axis. The length of the fragments of Aβ40 and Aβ42 fibrils was increased 4-fold by copying and subsequent parallel translation of the original short fragment along the central axis of the fibril, at a distance multiple of the distance between the two fibril layers. The resulting fibril fragments consisted of twelve layers and included 36 and 24 Aβ40 and Aβ42 molecules, respectively. The protonation state of the models was calculated at pH 7.4 and ionic strength 0.15 using the H++ web server (http://biophysics.cs.vt.edu/index.php; accessed on 1 September 2021; [86]). The net negative charge of fibril fragments atomic models was −108 for Aβ40 and −72 for Aβ42. Further, according to the protocol described below, the constructed fibril fragments were relaxed by 100 ns molecular dynamics simulations in an octahedral box filled with explicit solvent taking into account periodic boundary conditions.

The Amber18 software package [87] was used to minimize the potential energy of hydrated fibril fragments and their complexes with ThT and to run molecular dynamics simulations. The last 25 ns of productive MD trajectories were used for structural cluster analysis of two obtained fibril fragments. The median structure of the most populated cluster was chosen to search potential binding sites of chemical compounds and subsequent docking of the ThT molecule at these sites.

The analysis of the resulting structures, search for potential binding sites, and ThT docking were performed using the ICM Pro 3.8 molecular modeling software package [88]. The flexible docking procedure was carried out according to the protocol described in detail by the authors of the program [88,89,90]. To study the interaction of ThT with two fibril fragments by docking methods, we used the previously constructed atomic model of the dye [91].

Based on the docking results, for each of the two models of fibril fragments, we constructed five complexes with ThT molecules, representing the possible variants of interaction with the dye in the fullest manner. The number of ThT molecules in the complexes arises from the symmetry of the fibril fragment relative to the central axis. In the constructed complexes, there were three ThT molecules per fragment of the Aβ40 fibril, and four per fragment of the Aβ42 fibril. The stability of the constructed complexes was tested by the MD simulations according to the protocol given below. The VMD program was used for the visualization and analysis of molecular dynamics trajectories [92]. Changes in a ThT position on the fibril surface during MD were monitored visually; for binding sites where ThT was retained for more than 6 ns, the ThT binding free energy was calculated by the MM/GBSA method (ionic strength of 150 mM, generalized Born approximation igb5 by Onufriev et al. [93]).

### 4.10. Molecular Dynamics Simulations

Simulations in the explicit solvent were carried out in Amber18 [87], with AMBER ff14SB force field accounting for protein part [94] and gaff2 for ThT. The structures of the amyloids’ fragments or their complexes with ThT were placed in an octahedral box of TIP3P water, with the box edge at least 18 Å away from the solute at all points. Na+ ions were added to neutralize the system. Molecular dynamics simulations were carried out according to a standard protocol, including the following: (1) two-stage minimization, in which the first stage affected predominantly coordinates of the solvent molecules while positions of protein/ThT atoms were restrained (force constant 1.0 (kcal/mol)/Å^2^), and the second stage affected entire system; (2) gradual heating of the system from 0 K to 300 K during 100 ps, keeping the protein/ThT atoms restrained (force constant 1.0 (kcal/mol)/Å^2^); (3) 1 ns equilibration of the system with attenuated restrains at protein/ThT atoms (force constant 0.1 (kcal/mol)/Å^2^), followed by 1 ns equilibration of the unrestrained system; (4) 100 ns or 200 ns productive run.

Langevin thermostat with collision frequency γ = 2 ps^−1^ was used for temperature control at 300 K. The constant pressure was maintained using the isotropic scaling option and Berendsen barostat with pressure relaxation time set to 2 ps. Long-range electrostatics were treated with the particle mesh Ewald method; non-bonded Coulomb and Lennard–Jones interactions were truncated at 9 Å. The molecular dynamics integration step was 2 fs. All covalent bonds involving hydrogen atoms were constrained by the SHAKE algorithm. Trajectories were processed with the *cpptraj* module of AMBER18.

### 4.11. Cell Viability Assessment

Cell viability was analyzed using cervical cancer (HeLa) and glioma (GL-6) human cell lines; cells were within 13 passages when the experiment was conducted. Both lines were purchased from the Collection of Vertebrate Cell Cultures, Institute of Cytology, RAS, Saint Petersburg. Immortalized cell lines are relatively resistant to aggregated Aβ and retain metabolic activity up to µM concentrations of mature amyloid fibrils, which prevented us from working with lower concentrations of amyloid. However, as we noted, a local increase in the concentration of amyloidogenic peptides is possible in vivo. In addition, we carried out a relative assessment of the similarity or difference of cytotoxicity for two types of morphologically and physically different Aβ40 and Aβ42 aggregates, which do not depend on the concentration of amyloid (was shown for two different amyloid concentrations in our experiments).

The toxicity was assessed by the MTT (Sigma-Aldrich, St. Louis, MO, USA) reduction inhibition assay based on the protocol described for the first time by Mosmann [95]. Briefly, cells were routinely cultured in DMEM-10% FBS supplemented with 50 μg/mL penicillin–streptomycin and 2 mM L-glutamine and kept in a 5% CO_2_ humidified incubator at 37 °C. For the assay of the fibrils’ influence on the cell’s vitality, they (60% confluence) were stripped from culture flasks with 0.25% trypsin-EDTA, washed with DPBS, and plated in 96-well coated culture plates at a density of 6000 viable cells/well in 100 µL of DMEM. The cells were incubated at 37 °C and 5% CO_2_ for 24 h and amyloid aggregates were administered to cells. After 24 h, DMEM was removed and cells were incubated for 3 h with 100 μL of DMEM without phenol red and FBS, containing 0.5 µg/µL MTT. Following the treatment, 100 μL of DMSO was added to each well and the samples were incubated at 37 °C to allow complete lysis. The absorbance values were determined at 595 nm with an automatic plate reader (Bio-Rad, Milan, Italy). The final absorption values were calculated by averaging 5 independent measurements of each sample and subtracting from this the average of the blank. Readings from the different conditions were expressed as percent activity concerning controls containing equal amounts of Aβ incubation buffer.

The results of the experiments were presented as median. All experiments were performed at least in triplicate. To test the sample data for normal distribution, the Kolmogorov–Smirnov test was used. Multiple group comparisons were processed using the one-way analysis of variance (ANOVA) method with Tukey’s post hoc test. The differences were considered significant at *p* < 0.05. Data were analyzed using online calculator software (https://astatsa.com/OneWay_Anova_with_TukeyHSD/, accessed on 1 December 2021).

To visualize live and dead cells, double fluorescence staining with propidium iodide (PI) and rhodamine 123 (Rh123) was used. These dyes are basically non-toxic and have been used as indicators of cell integrity and cell viability. PI is a polar compound that enters cells with damaged cell membranes, binding to nucleic acids, and Rh123 accumulates in membranes of living cells. Briefly, cells (8000 cells/cm^2^) were plated on 12-well coated plates (Biofil, China) and treated with amyloid samples (5 µM by monomer equivalent) in DMEM for 24 h. Then cells were washed with phosphate-buffered saline (pH 7.4), Rh123 was added to a final concentration of 5 µg/mL, and the samples were incubated for 5 min at 37 °C in the dark. The cells were next washed with dye-free PBS to eliminate non-specific binding, and PI (Sigma, St. Louis, MO, USA) was added to a final concentration of 5 µg/mL. The samples were incubated for 5 min at 37 °C in the dark and washed again.

Acquisition of fluorescent images was carried out using an FV3000 microscope (Olympus, Japan) with LUCPLFLN 40X objective and laser set (ex. 488 nm and em. 510 nm for RH 123 and ex. 561 nm and em. 603 for PI). Images were directly obtained in digital format as TIFF files with a resolution of 1024 × 1024 pixels.

### 4.12. Statistical Analysis

The photophysical characteristics of amyloids and fibril-bound ThT were determined from at least three independent experiments. The standard error of the mean was determined for a confidence interval of 0.95. The reliability of the results was verified according to a one-way analysis of variance (ANOVA) using GraphPad Prism (Version 9.1.0) software.

## Figures and Tables

**Table 1:** Parameters of ThT binding to amyloid fibrils formed from Abeta peptides and other amyloidogenic proteins.

Object	λ_max_, nm	i	K_bi_ × 10^−5^, M^−1^	n_i_	q_i_
ThT bound to Aβ40 amyloid fibrils	441	1	0.25 ± 0.07	0.12 ± 0.05	0.12 ± 0.01
ThT bound to Aβ42 amyloid fibrils	432	1	0.17 ± 0.05	0.27 ± 0.05	0.010 ± 0.001
		2	70 ± 5	0.005 ± 0.003	0.25 ± 0.01
ThT bound to insulin amyloid fibrils [40]	450	1	0.35	0.14	0.27
		2	78	0.02	0.72
ThT bound to lysozyme amyloid fibrils [33]	449	1	0.6	0.25	0.0001
		2	72	0.11	0.44
ThT bound to beta-2-microglobulin amyloid fibrils [34]	442	1	0.34 ± 0.04	0.041 ± 0.006	0.36 ± 0.03
Free ThT in aqueous solution [41]	412	-	-	-	0.0001

i—number of binding modes; K_bi_ and n_i_—the binding constant and the number of ThT binding sites with the i mode of fibrils, respectively; λ_max_ and q_i_—the wavelength of the absorption spectrum maximum and the fluorescence quantum yield of ThT bound to the i mode of the fibrils.

## Data Availability

The data that support the findings of this study are available from the corresponding author upon reasonable request.

## Supplementary Materials

The following are available online at https://www.mdpi.com/article/10.3390/ijms23105513/s1.
