# Supplementary material for: New Evidence on a Distinction between Aβ40 and Aβ42 Amyloids: Thioflavin T Binding Modes, Clustering Tendency, Degradation Resistance, and Cross-Seeding
Source: Int J Mol Sci. 2022 May 15;23(10):5513. doi: 10.3390/ijms23105513 (PMC9141448; doi:10.3390/ijms23105513)
Supplement: Supplementary file 1 [file ijms-23-05513-s001.zip › ijms-1701700-supplementary.pdf]

Supporting Information

New Evidence on a Distinction between Aβ40 and Aβ42 Amyloids: Thioflavin T Binding Modes, Clustering Tendency, Degradation Resistance, and Cross-Seeding

Anna I. Sulatskaya[1], Georgy N. Rychkov[2,3], Maksim I. Sulatsky[4], Ekaterina V. Mikhailova[1], Nadezhda M. Melnikova[1], Veronika S. Andozhskaya[1], Irina M. Kuznetsova[1], Konstantin K. Turoverov[1,*]

[1] Laboratory of structural dynamics, stability and folding of proteins, Institute of Cytology, Russian Academy of Sciences, 4 Tikhoretsky ave., 194064 St. Petersburg, Russia; ansul@mail.ru (A.I.S.); 4evamkh@gmail.com (E.V.M.); melnikova07nm@gmail.com (N.M.M.); veronika.andozhskaya@gmail.com (V.S.A.); imk@incras.ru (I.M.K.); kkt@incras.ru (KKT)

[2] Laboratory of Molecular and Cellular Biophysics, Petersburg Nuclear Physics Institute Named by B.P. Konstantinov of National Research Center «Kurchatov Institute», mkr. Orlova roshcha 1, 188300 Gatchina, Russia; georgy-rychkov@yandex.ru (G.N.R.)

[3] Institute of Biomedical Systems and Biotechnology, Peter the Great St. Petersburg Polytechnic University, Polytechnicheskaya 29, 195251 St. Petersburg, Russia;

[4] Laboratory of cell morphology, Institute of Cytology, Russian Academy of Sciences, 4 Tikhoretsky ave., 194064 St. Petersburg, Russia; m_sulatsky@mail.ru (M.I.S.)

* Correspondence: ansul@mail.ru (A.I.S.); imk@incras.ru (I.M.K.); kkt@incras.ru (K.K.T.); Tel.: +7-812-297-19-57

Table of Contents

Amino acid sequence of Abeta-peptides (Scheme S1)...S-3

Distribution of the dihedral angle between the aminobenzene and benzthiazole rings of ThT located in the binding sites of Aβ40 (A) and Aβ42 (B) fibrils (Figure S1) S-4

Fluorescence intensity of thioflavin T (ThT) probe in the presence of GdnHCl (Figure S2)..... S-5

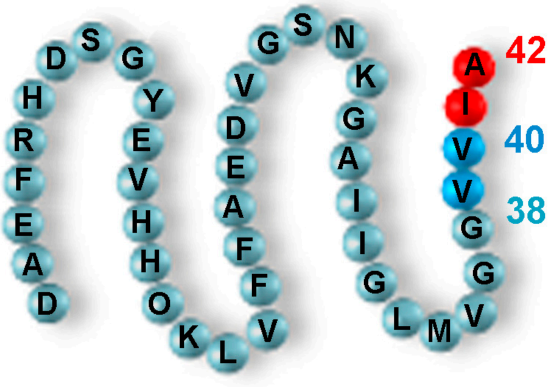

Scheme S1. Amino acid sequence of Abeta-peptides.

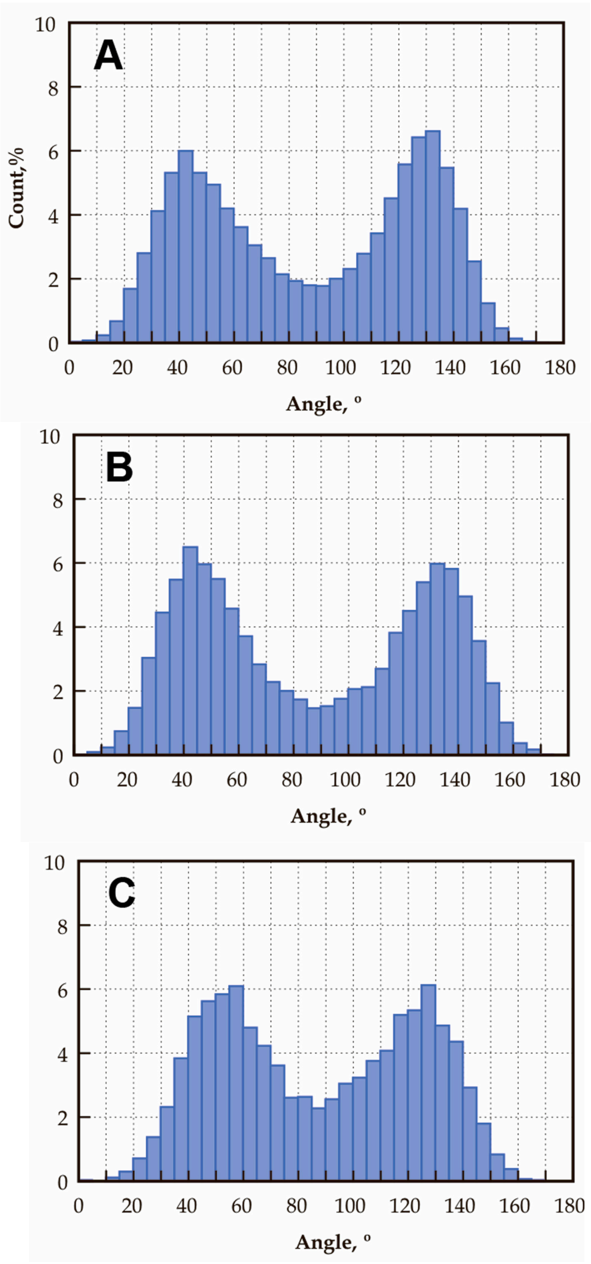

Figure S1. Distribution of the dihedral angle between the aminobenzene and benzthiazole rings of ThT located in the binding sites of Aβ40 (**A**) and Aβ42 (**B**) fibrils, characterized by the lowest free binding energy, as well as ThT in the free state in an aqueous environment (**C**).

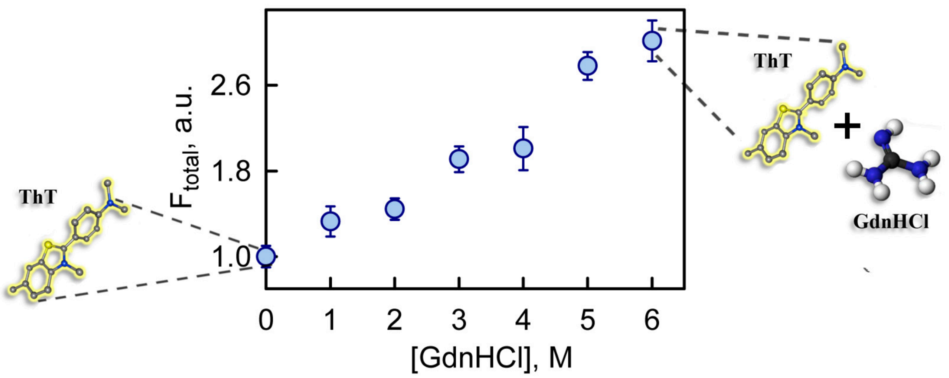

Figure S2. Fluorescence intensity of thioflavin T (ThT) probe in the presence of GdnHCl. Total ThT fluorescence intensity normalized to fluorescence of free dye in water solution is presented.
